# Using the Spleen as an *In Vivo* Systemic Immune Barometer Alongside Osteosarcoma Disease Progression and Immunotherapy with *α*-PD-L1

**DOI:** 10.1155/2018/8694397

**Published:** 2018-12-12

**Authors:** Justin E. Markel, Jabeen Noore, Eric J. Emery, Harley J. Bobnar, Eugenie S. Kleinerman, Brock A. Lindsey

**Affiliations:** ^1^Department of Orthopaedics, West Virginia University, Morgantown, WV, USA; ^2^School of Medicine, West Virginia University, Morgantown, WV, USA; ^3^Washington and Jefferson College, Washington, PA, USA; ^4^The University of Texas MD Anderson Cancer Center, Houston, TX, USA

## Abstract

Indications for immunotherapies are still unclear, and there is a great need for real-time patient immune status monitoring. In this study, we confirmed that the local and systemic immune profiles of an orthotopic osteosarcoma model with or without luciferase transfection were statistically equivalent. Next, we used flow cytometry to describe systemic immune cell populations influenced by osteosarcoma disease progression. When compared to vehicle-inoculated sham mice, it was found that tumor-bearing mice had significant immunophenotype disturbances at approximately 11 weeks after inoculation (at which time 90% of primary tumor-bearing mice have fulminant pulmonary metastases). Percent populations of natural killer cells and T regulatory cells were increased in the spleens of tumor-bearing mice (*p* < 0.0083) compared to shams. Additionally, T lymphocytes from spleens of tumor-bearing mice showed increased Tim-3/PD-1 exhaustion status (*p* < 0.0083). There were also increases in the percent populations of myeloid cells and overall M1/M2 macrophage marker expression on tumor-bearing mice spleens versus controls (*p* < 0.00714). Finally, treatment with 20 *μ*g *α*-PD-L1 decreased T-cell exhaustion back to sham status, with a corresponding increase in CTLA-4 expression on cytotoxic T cells in the majority of mice tested. Checkpoint inhibition also increased splenic monocyte maturation and returned macrophage M1/M2 marker expression back to sham status. These data suggest that cancer induces systemic immune dysregulation and that these changes may be elucidated and utilized for treatment purposes by sampling the systemic immune environment via the spleen. In addition, treatment with the checkpoint inhibitor *α*-PD-L1 may neutralize but not overcome the systemic immunological changes induced by a progressing malignancy.

## 1. Introduction

Osteosarcoma is an extremely aggressive tumor of bone, and no new therapeutics have been implemented clinically in over three decades [1]. Its extreme pleomorphism and high antigenic load [2, 3] make it a seemingly prime candidate for tumor immunotherapies, which can induce sustainable immunity against cancer cell neoantigens, thus providing a viable therapeutic strategy for overcoming heterogeneity; however, no immunotherapies are currently implicated as standard of care in the treatment of osteosarcoma. As the malignant process can lead to generalized immune dysfunction and disease progression [4–6], there has been success across multiple tumor types with the introduction of various immunotherapies into systemic circulation including monoclonal antibody checkpoint blockers like those against programmed death-ligand 1 (PD-L1) and its receptor, programmed cell death protein 1 (PD-1) [7]. PD-L1 is expressed on tumor and antigen presenting cells (APCs), while its receptor PD-1 is expressed on activated T cells; this interaction suppresses T-cell activity and is associated with T-cell exhaustion (TCE), a state of depressed effector function and anergy brought on by chronic antigen exposure and hypoxia [8]. Osteosarcoma tumors are often positive for PD-L1, and its expression has been shown to upregulate in metastatic versus primary lesions [9]. While blockade of this protein interaction has experienced clinical success in some cancers, there is still confusion surrounding its specific indications and only a minority shows a response [10]; preclinical mouse models of metastatic osteosarcoma have shown resistance to PD-L1/PD-1 blockade [11]. Moreover, there is currently no reliable way of monitoring or predicting patient responses to any immunotherapies including checkpoint blockers, and the search for relevant biomarkers is ongoing [12]. Indeed, data of this nature would potentially allow clinicians to adjust regimens as necessary for maximal efficacy in real-time and may uncover reasons as to why tumors like osteosarcoma have remained largely unresponsive to immunotherapies despite their high neoantigen load. From the few studies currently available on osteosarcoma tumor immunology, data have emerged that support the importance of macrophage M1/M2 polarization and T-cell exhaustion and activation in tumor clearance, although the scope has generally been limited to the primary lesions [11, 13–21]. We suggest that these data warrant further investigation regarding the translation of macrophage-T cell dynamics systemically and how we might use this information clinically to trigger antitumor immunity in patients. In the present study, after confirming immunologic equivalency between two models of osteosarcoma (with or without introduction of a nonviral luciferase reporter vector for *In Vivo* Imaging System (IVIS) visualization), we further defined the immunological consequences of osteosarcoma disease progression over time. We also investigated the systemic effects of monotherapy with checkpoint blockade of PD-L1 using the spleen as a barometer of immune status. The overarching hypothesis is that the spleen can be used as a barometer to assess clinically relevant changes in the systemic macrophage-T cell immunophenotype brought on by osteosarcoma disease progression and immunotherapy.

## 2. Materials and Methods

### 2.1. Animals

Female BALB/c mice aged 4-5 weeks and between 20 and 25 grams (mean = 22.5 grams) in mass were obtained from The Jackson Laboratory (Bar Harbor, ME). Mice were housed individually in ventilated Allentown cages at ambient temperatures within specific pathogen-free facilities on corncob bedding with 12 hour light/dark cycles, automatic lixit water, and ad libitum food access. All experiments were approved by the Institutional Animal Care and Use Committee.

### 2.2. Growth and Preparation of Transfected and Wild-Type K7M2 Cells

K7M2 cells were grown and prepared as previously described [22]. Briefly, wild-type (WT) and genome-stable luciferase-transfected (TF) K7M2 cells were cultured in Dulbecco's Modified Eagle's Medium (DMEM) containing 10% fetal bovine serum (FBS) and 100 units/mL penicillin and streptomycin (Thermo Fisher Scientific, Waltham, MA). Passage one cells were used for orthotopic implantation at a density of one million cells in 25 *μ*L of phosphate buffered saline (PBS) without calcium or magnesium (Corning Inc., Corning, NY).

### 2.3. Osteosarcoma BALB/c K7M2 Syngeneic Mouse Model

K7M2 cells or vehicle were surgically inoculated into the tibias of female BALB/c mice as previously described [22]. Briefly, 25 *μ*L of PBS containing one million TF or WT K7M2 cells were dispensed into a cortical window made through the center of the tibia at the level of the proximal tibial flare.

WT and TF tumor-bearing mice were monitored as previously described [22] in accordance with our institution's tumor burden scoring system. Tumors were monitored daily using digital calipers (greatest width × greatest length). Tumor volume was calculated using the formula V = (length × width^2^)/2. The health of each animal was assessed using a tumor score ranging from 0 to 60 (0 = healthy animal; 60 = requires euthanasia) based on criteria established by our institution regarding overall animal health as previously described [22]. Once mice reached a tumor score of 60 (approximately 11 weeks after inoculation), animals were humanely euthanized using carbon dioxide asphyxiation.

Tumors were harvested as previously described [22]. Briefly, animals whose tumors reached an average of 1.7 cm^3^ underwent palliative amputation by dissection and hip disarticulation. Skin edges were closed using 4-0 vicryl suture. Tumors were dissociated and prepared for flow cytometry analysis as described below. Once tumor scores reached 60 (approximately 11 weeks after inoculation), animals were euthanized and their spleens harvested and prepared for flow cytometry (FC) analysis.

### 2.4. Flow Cytometry Analysis of Tumors and Spleens

The excised primary tumor and spleen from each mouse were minced and digested enzymatically using the MACS Tissue Dissociation Kits (Miltenyi Biotec, Auburn, CA), according to the manufacturer's guidelines. After dissociation, each sample was filtered through a 70 *μ*M filter (Miltenyi Biotec) and centrifuged at 300 ×g for 10 min; the cell pellet was resuspended in RPMI-1640. Each single-cell suspension was split into two. One half of the tumor single cell suspension was treated with Red Blood Cell Lysis Solution (Miltenyi Biotec), washed twice, and counted.

Spleen single-cell suspensions and the other half of the tumor single cell suspensions were layered over Ficoll-Paque premium (GE Healthcare Life Sciences, Pittsburgh, PA) and centrifuged at 400 ×g for 40 min at 20°C to allow density gradient separation of the leukocytes. After Ficoll, the leukocytes (buffy coat) were harvested, washed twice with PBS, and counted.

Single-cell suspensions were stained following standard protocols. Briefly, cells were washed using FACs buffer (PBS, 2% FBS, and 0.02% sodium azide). Cell pellets were resuspended, blocked using Rat IgG and Mouse IgG (Jackson ImmunoResearch, West Grove, PA), washed, and incubated with the appropriate antibodies described below. After incubation, the cells were washed, fixed in paraformaldehyde, and stored at 4°C.

#### 2.4.1. Panel 1 Antibodies

*α*-CD45-phycoerithrin cyanine5 (BD Biosciences, San Jose, CA), *α*-CD4-BV510 (BD Bioscience), *α*-Ly6G-V450 (BD Biosciences), *α*-CD8a-Alexa Fluor 488 (BD Biosciences), *α*-NKp46-PE-eFluor 610 (eBioscience, San Diego, CA), *α*-CD11b-Alexa Fluor 700 (eBioscience), and *α*-Foxp3-phycoerithrin (eBioscience)

#### 2.4.2. Panel 2 Antibodies

*α*-CD45-phycoerithrin cyanine5 (BD Biosciences) and *α*-PD-L1-phycoerithrin cyanine7 (eBioscience)

#### 2.4.3. Panel 3 Antibodies

*α*-CD45-phycoerithrin cyanine5 (BD Bioscience), *α*-CD4-BV510 (BD Bioscience), *α*-CD8a-Alexa Fluor 488 (BD Bioscience), *α*-Tim-3-PerCP/Cy5.5 (BioLegend, San Diego, CA), *α*-PD-1-eFluor 450 (Thermo Fisher Scientific), *α*-CTLA-4-PE/Cy7 (BioLegend), and *α*-Foxp3-phycoerithrin (eBioscience)

#### 2.4.4. Panel 4 Antibodies

*α*-CD45-phycoerithrin cyanine5 (BD Biosciences), *α*-CD11b-Alexa Fluor 700 (eBioscience), *α*-Ly6G-V450 (BD Bioscience), *α*-Ly6C-PerCP/Cy5.5 (eBioscience), *α*-CXCL9-phycoerithrin (eBioscience), *α*-NOS2-PE-cyanine7 (BioLegend), *α*-Tgm2-Alexa488 (Novus Biologicals, Littleton, CO), and *α*-Arg1-APC (R&D Systems, Minneapolis, MN)

All cell populations and/or pertinent markers as they are used in this manuscript are defined in Table 1.

For Foxp3 staining, after washing cells with FACS buffer, the cells were processed using a Foxp3/transcription factor staining buffer set (eBioscience) according to the manufacturer's instructions. Briefly, the cell pellet was resuspended and 1.0 mL of diluted (1:3 ratio) Foxp3 fixation/permeabilization buffer was added. Samples were vortexed and incubated at 4°C overnight. The next day, the cells were washed twice with 1X Foxp3 buffer and incubated with *α*-Foxp3-phycoerithrin (eBioscience), washed with 1X Foxp3 buffer, and resuspended in FACS buffer.

Panel 4 included intracellular staining of M1/M2 macrophage markers. For this panel, after washing cells with FACS buffer, the cells were further processed for intracellular antigens using the intracellular antigen staining buffer set (BioLegend) according to the manufacturer's instructions. Briefly, cell pellets were resuspended, and 100 *μ*L of intracellular-fixation buffer was added. After a pulse vortex, the cells were incubated at 4°C overnight. The next day, the cells were washed twice with 1X permeabilization buffer, incubated with a master-mix of *α*-CXCL9-phycoerithrin (eBioscience), *α*-NOS2-PE-cyanine7 (BioLegend), *α*-Tgm2-Alexa488 (Novus Biologicals), and *α*-Arg1-APC (R&D Systems), washed twice with 1X permeabilization buffer, and resuspended in FACS buffer.

Cells were analyzed, and the data were collected on the BD LSRFortessa (BD Biosciences) using BD FACS Diva version 8.0 software located in the Flow Cytometry & Single Cell Core Facility. Single stained controls were generated using OneComp beads eBeads (eBiosciences). A minimum of 50,000 cells were analyzed for each sample. Data analysis was performed using FCS Express 6 Software (De Novo Software, Glendale, CA).

### 2.5. T-Cell Exhaustion Status (TCES)

T cells expressing the exhaustion markers PD-1 and/or T-cell immunoglobulin and mucin-domain containing-3 (Tim-3) exhibit depressed effector functions and an inability to proliferate [23–25]; exhausted Tim-3^+^ and PD-1^+^ T cells have been found in osteosarcoma tumor tissues [26]. In order to properly compare expression of these TCE markers on the T-cell repertoire across mice with different cell counts, we normalized for total T-cell percent population to yield an overall exhaustion status we refer to as the TCE status (TCES) calculated by the following equation:

(1) $$\begin{array}{c} \text{TCES}=\frac{\text{total}\%\text{PD}1\;\;{\text{expression}}_{\text{CTL, Th}}+t\text{otal}\%{\text{Tim}3}_{\text{CTL, Th}}}{\text{total}\%\text{CTL}+t\text{otal}\%\text{Th}}. \end{array}$$

In words, the TCES equals the total PD-1 and Tim-3 percent expression on both CD4^+^ T helper cells (Th) and CD8^+^ cytotoxic T cells (CTLs) divided by the total percent population of all T cells (both CD4^+^ and CD8^+^) sampled. This equation yields the ratio of exhaustion-marker expressing T lymphocytes per total lymphocyte percent population and is driven by peer-reviewed data showing that PD-1 and Tim-3 expression on T cells correlates with the extent of TCE [23, 27–29]. Normalizing for total T-cell percent populations allows comparisons to be made across multiple different samples with varying T-cell counts.

### 2.6. *α*-PD-L1 Treatment

Mice were given 20 *μ*g (1 mg/kg) *α*-PD-L1 (clone MIH5, eBioscience) via intraperitoneal injection every Monday and Thursday starting when tumors were first palpable and continuing until euthanasia (approximately 11 weeks following tumor cell inoculation). The standard dosing used throughout the literature is 200 *μ*g (10 mg/kg); however, 1 mg/kg is the dosage at which clinical activity in advanced human cancer starts [7], although extended treatment periods with doses as high as 20 mg/kg have been shown ineffective against metastatic K7M2 tumors [11]. For this manuscript, which focuses purely on the immunological changes associated with PD-L1 blockade, the minimum dose needed to produce a measurable immune response (1 mg/kg) was used as there is no known *α*-PD-L1 dose that produces clinical benefit in osteosarcoma.

### 2.7. Statistical Analysis

Statistical significance between groups was assessed using a Student's *T*-test with alpha = 0.05 and Bonferroni correction. A minimum of three animals per group were used, which is a widely used starting number and allows for the determination of potential outliers.

## 3. Results and Discussion

### 3.1. The Local and Systemic Immune Environments of BALB/c Mice Orthotopically Inoculated with Luciferase(+) TF or Luciferase(−) WT K7M2 Cells Are Statistically Equivalent

Our laboratory previously developed an orthotopic model of metastatic osteosarcoma using BALB/c syngeneic K7M2 tumor cells transfected with a luciferase reporter for real-time disease monitoring using IVIS. This model allows for sensitive visualization and assessment of primary and metastatic lesions without euthanasia in an immunocompetent mouse. From a clinical outcomes perspective, both luciferase(+) TF and luciferase(−) WT models were previously shown to result in statistically equivalent rates of primary lesion formation and pulmonary metastasis [22]. However, as luciferase is a foreign intracellular protein under a constitutively active promoter, it is presented on MHC class I molecules [30] and has the potential to elicit atypical immune reactions [31, 32]. As this manuscript focuses exclusively on characterizing antitumor immune responses, we considered it extremely important to first examine whether the luciferase antigen significantly affected the immune microenvironment. To accomplish this goal, we assessed the local (tumors harvested at approximately 1.7 cm^3^ in volume) and systemic (splenic) immune microenvironments by flow cytometry. The spleen is a large lymph node-like immune organ with direct access to blood-borne pathogens and antigens and thus a good representation of systemic immune function.

To determine if the local tumor immune microenvironments were influenced by the presence of the luciferase antigen, we surveyed a variety of key myeloid and lymphoid lineage immune cells (six populations in total) with maximal clinical relevance in both WT tumor-bearing (*n* = 11) and TF tumor-bearing (*n* = 15) mice using Antibody Panel 1. The populations investigated included CD45^+^CD8^+^ CTLs, CD45^+^CD4^+^ Ths, CD45^+^CD4^+^Foxp3^+^ T regulatory cells (Tregs), CD45^+^NKp46^+^ natural killer (NK) cells, CD45^+^CD11b^+^ myeloid lineage cells (MLCs), and CD45^+^CD11b^+^Ly6G^+^ granulocytes. A sample gating schema is shown in Supplementary Figures 1–3. Using the Bonferroni correction, individual comparisons were made using *α* = 0.0083 and all populations were deemed statistically equivalent (*p* > 0.0083) between the two groups (Table 2). Additionally, as PD-L1 expression in tumors has been speculated to play an important part in osteosarcoma-mediated immune suppression [33, 34], we also examined whether PD-L1 expression differed between groups. We looked for expression of PD-L1 on tumor cells using the CD45^−^ gate (which identified all nonleukocyte populations within the tumor) in Antibody Panel 2; a sample gating schema is shown in Supplementary Figure 4. No statistical difference (*p* > 0.05) was observed in percent PD-L1 expression on tumors generated from both TF tumor-bearing (*n* = 15) and WT tumor-bearing (*n* = 11) mice (Table 3).

To further evaluate the immunological equivalence between TF and WT models, we sought to determine whether the systemic immune microenvironments were influenced by the presence of luciferase. Previous data had shown that approximately eleven weeks following tumor cell inoculation, 90% of mice in both TF and WT tumor-bearing groups developed pulmonary metastases [22]. At this time, mice were euthanized and splenocytes were immunophenotyped with Antibody Panel 1 to determine if splenic immunomodulation in response to late-stage disease was consistent between groups. Using the Bonferroni correction, individual comparisons were made using *α* = 0.0083 and like that for primary tumors, the splenic microenvironments of mice from both groups were almost uniformly statistically equivalent (*p* > 0.0083), except for the percentage of granulocytes (WT_gran_ = 15.27 ± 21.82%: *n* = 10; TF_gran_ = 52.33 ± 19.02%: *n* = 9; *p*=0.0011). The statistics for these analyses are shown in Table 4.

Whenever a foreign reporter protein is introduced into *in vivo* systems, there is the potential for it to interfere with normal immune processes; however, this aspect of tumor immunology is often overlooked in the literature even though it has the potential to invalidate conclusions. As this manuscript focuses on elucidating the antitumor responses, we provide evidence to support that introduction of a luciferase reporter did not dramatically alter the antitumor immune response. The white blood cell (WBC) populations examined in Panel 1 presented a broad overview of key mediators of tumor immunology including NK cells, CTLs, Ths, Tregs, and MLCs. Overall, out of the 13 comparisons made between TF and WT tumor-bearing models, only one population (percent CD45^+^CD11b^+^Ly6G^+^ granulocytes in the spleen) was deemed statistically inequivalent. However, we dismissed it as the probable consequence of a type 1 error at the 95% confidence interval and concluded that constituent expression of the luciferase reporter did not significantly affect the local (tumor) or systemic (splenic) immune responses. Therefore, we continued our study of the systemic immune response to osteosarcoma using only the TF luciferase(+) model which allows for bioluminescent detection and quantification of disease burden.

### 3.2. Using the Spleen to Identify the Baseline Systemic Immune Fingerprint Associated with Disease Progression and Metastasis in the Luciferase(+) K7M2 Orthotopic BALB/c Mouse Model of Osteosarcoma

There is a critical gap in knowledge regarding the effects of malignancy on a patient's systemic immune status, especially in the context of tumor immunotherapy. Many immunotherapies (including monoclonal antibody checkpoint blockers) used in both preclinical and clinical settings are given systemically via the blood and can modulate interactions between T cells and APCs like macrophages [35–39]; however, our understanding of what systemic immunomodulations underlie tumor clearance (or resistance) is incomplete. Immunotherapies targeting this axis have shown great antitumor potential and, for the case of Mifamurtide, come the closest to regular clinical use in osteosarcoma [40]. Additionally, there is currently no reliable way to monitor immune activation changes over time once primary tumors are removed. Therefore, we sought to establish a baseline systemic immunological profile for the TF tumor-bearing mouse model that can be used by other investigators to examine tumor and/or treatment-induced immunomodulation. The spleen was chosen as a marker of systemic immune status as it is a central hub for circulating immune cells (including mature macrophages) and is the main filter for blood-borne antigens (including circulating tumor cells and debris). From a feasibility and clinical translational standpoint, splenic biopsies performed with fine-needle aspirates are an effective cytological monitoring platform with low complication rates in humans [41]. At 11 weeks after inoculation (at which time 90% of tumor-bearing mice have fulminant pulmonary metastases), we used Antibody Panels 3 and 4 to monitor the cellular changes of lymphoid lineage cells (LLCs) and MLCs, respectively, occurring alongside disease progression in the spleens of TF tumor-bearing mice. Antibody Panel 3 examined the following four LLC subsets of WBC populations: CD45^+^CD8^+^ CTLs, CD45^+^CD4^+^ Ths, CD45^+^CD4^+^Foxp3^+^ Tregs, and CD45^+^NKp46^+^ NK cells (example gating schema seen in Supplementary Figure 5). We also investigated the exhaustion status of the CD4^+^ and CD8^+^ T-lymphocyte populations using the prototypical cell-surface markers PD-1 and Tim-3. TCE is a phenomenon characterized by decreased effector cell function first described in the instance of chronic viral infection that correlates with increases in the expression levels of PD-1 and Tim-3 [23, 27–29]. Indeed, it has been shown that, like viral infections, cancers can cause similar immunological disruptions in lymphocyte function [42]. An additional T-cell surface molecule included in the LLC panel is cytotoxic T lymphocyte-associated protein 4 (CTLA-4) which competes with CD28 for binding to the B7 molecules CD80 and 86 on APCs inhibiting CTL function can also play a role in the immunological response to cancer [43]. An example gating schema for PD-1, Tim-3, and CTLA-4 expression on T cells can be seen in Supplementary Figure 6. The expression profile data for Antibody Panel 3 from the spleens of five mice are shown in the left column of Table 5. In summary, NK cell percent population averaged 10.78 ± 4.5%; percent populations of CTLs, Ths, and Tregs averaged 2.73 ± 1.22%, 6.45 ± 3.08%, and 21.09 ± 5.12%, respectively. CTLA-4 expression on CTLs and TCES averaged 18.35 ± 10.12% and 18.23 ± 4.58%, respectively.

Antibody Panel 4 investigated the MLC component of the splenic immunophenotype. The three cell populations examined included CD45^+^CD11 b^+^ MLCs, CD45^+^CD11 b^+^Ly6G^−^Ly6C^+^ monocytes/macrophages, and CD45^+^CD11 b^+^Ly6C^−^Ly6G^+^ granulocytes (example gating schema in Supplementary Figure 7). Additionally, the expression of the following four macrophage polarization markers transglutaminase 2 (Tgm2), arginase 1 (Arg1), chemokine (C-X-C motif) ligand 9 (Cxcl9), and nitric oxide synthase 2 (Nos2) was also assessed (example gating schema in Supplementary Figure 8). Tgm2/Arg1 and Cxcl9/Nos2 are classical M2-and M1-like macrophage markers typically associated with pro-tumor anti-inflammatory or antitumor pro-inflammatory properties, respectively. CD45^+^CD11b^+^ MLCs were separated into Ly6C^+^Ly6G^−^ monocytes and Ly6G^+^Ly6C^−^ granulocytes. From there, the total percent expression of the following four M1/M2 macrophage markers on Ly6C^+^Ly6G^−^ monocytes was determined, and the expression profile data for these markers from the spleens of five mice is shown in the left column of Table 6. In summary, MLC, monocyte/macrophage, and granulocyte percent populations averaged 5.27 ±1.11%, 4.92 ± 1.29%, and 57.49 ± 9.62%, respectively. Percent expression of individual M1/M2 markers Tgm2, Arg1, Cxcl9, and Nos2 on macrophages was 18.58 ± 5.69%, 0.3 ± 0.56%, 18.28 ± 5.38%, and 97.99 ± 1.62%, respectively.

Here, we have developed two in-depth antibody panels (Panels 3 and 4) that can be used to assess the splenocyte immune fingerprint. The data gathered can be used as a baseline by which to assess immunomodulation in response to therapeutic intervention. To date, the splenic lymphoid and myeloid immune fingerprints of osteosarcoma tumor-bearing mice at the time of pulmonary metastasis have never been investigated. Note that, in this study, macrophages were not classified overtly as M1 or M2; rather, we report only the percent expression of each individual marker expressed by the entire macrophage population. As opposed to studies that choose the presence or absence of a single marker to identify M1/M2 polarization, our paradigm provides a more objective view of macrophage phenotypes as complex disease processes like cancer influence macrophages to take on intermediate phenotypes and co-express both M1 and M2 markers [44, 45]. Analyzing the percent expression of each individual marker on the macrophage population allows for the full spectrum of macrophage phenotypes to be appreciated, compared with using single markers to incorrectly generalize and simplify a complex population.

### 3.3. Osteosarcoma Disease Progression and Metastasis Induce Systemic Immunomodulation as Visualized by Splenocyte Immunophenotyping

**Figure 1:**
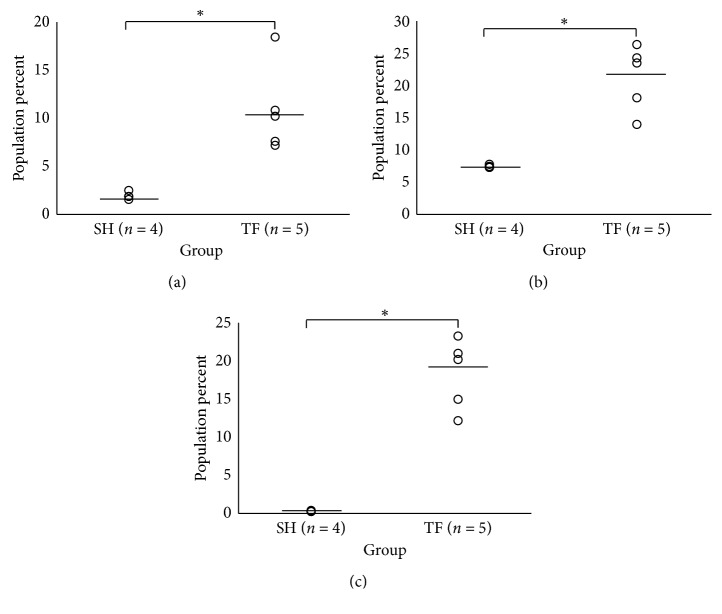
Osteosarcoma induces systemic immunomodulation in splenic LLC populations at 11 weeks. The spleens of TF tumor-bearing (*n* = 5) and SH (*n* = 4) mice were harvested at time of metastatic disease (approximately 11 weeks) after inoculation and analyzed via flow cytometry for LLC immunophenotyping. The data spread of the following percent populations (a) CD45^+^NKp46^+^ NK cells and (b) CD45^+^CD4^+^Foxp3^+^ Tregs between groups are displayed. (c) Data spread of TCES (total PD-1 and Tim-3 expression normalized to total percent T-lymphocyte population) for each group. An asterisk denotes statistical significance (*p* < 0.0083) between groups as determined by a two-tailed Student's *T*-test with Bonferroni correction and 95% confidence. LLC: lymphoid lineage cell; TF: transfected; SH: sham; NK: natural killer; Treg: T regulatory cell; TCES: T-cell exhaustion status; PD-1: programmed cell death protein 1; Tim-3: T-cell immunoglobulin and mucin-domain containing-3.

**Figure 2:**
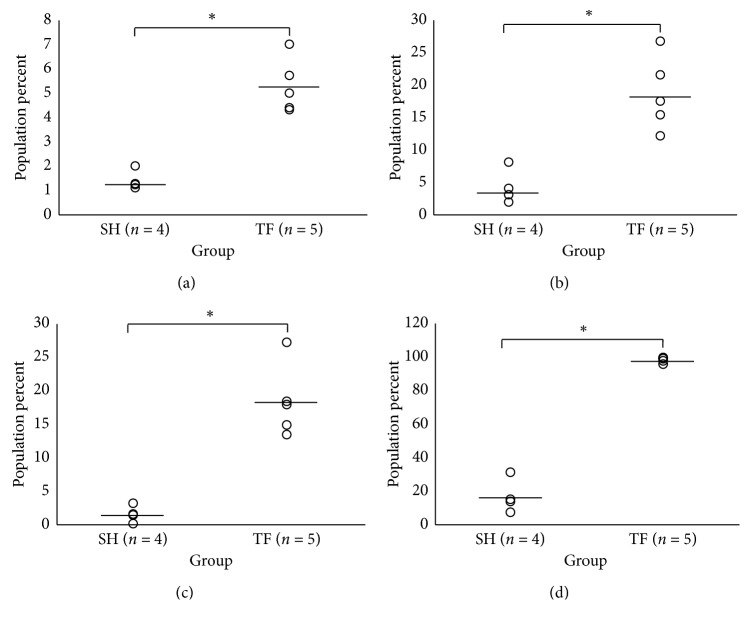
Osteosarcoma induces systemic immunomodulation in splenic MLC populations at 11 weeks. The spleens of TF tumor-bearing (*n* = 5) and SH (*n* = 4) mice were harvested at time of metastatic disease (approximately 11 weeks) after inoculation and analyzed via flow cytometry for MLC immunophenotyping. The data spread of the percent parent populations (a) CD45^+^CD11 b^+^ MLCs is shown. Also, the spreads of an M2 macrophage polarization marker (b) Tgm2 and two M1 macrophage polarization markers (c) Cxcl9 and (d) Nos2 on CD45^+^CD11 b^+^Ly6C^+^Ly6G^−^ monocytes/macrophages are shown. An asterisk denotes statistical significance with *p* value < 0.00714 from a two-tailed Student's *T*-test with Bonferroni correction and 95% confidence. MLC: myeloid lineage cell; TF: transfected; SH: sham; Tgm2: transglutaminase 2; Cxcl9: chemokine (C-X-C motif) ligand 9; Nos2: nitric oxide synthase 2.

Evidence suggests that both lymphoid and myeloid lineage activation status in both the local and systemic immune environments may carry clinically useful information regarding osteosarcoma disease progression, metastasis, and survival [16, 17, 46]. However, once the primary tumor is removed, it can no longer provide up-to-date information on the evolving antitumor immune response. Peripheral blood has been used to determine immune status outside of the primary tumor with promising results in other cancers [47–51]; however, the story is incomplete as mature macrophage data cannot typically be obtained in this fashion. Therefore, we sought to determine if the spleen can be used to detect tumor-induced immunomodulation by comparing the spleens of TF tumor-bearing mice to those undergoing sham (SH) surgery procedures to examine the extent of systemic splenic immunomodulation induced by malignancy. SH mice underwent the same surgical procedures and Antibody Panels 3 and 4 as tumor-bearing mice; however, they were inoculated with vehicle as opposed to tumor cells. Using the Bonferroni correction, individual comparisons were made between TF tumor-bearing (*n* = 5) and SH (*n* = 4) mice via Student's *T*-test using *α* = 0.0083 and *α* = 0.00714 for Antibody Panels 3 and 4, respectively, to assess significance at the 95% confidence interval. Between the two panels, numerous statistically significant differences between TF tumor-bearing and SH mice were discovered. For Panel 3, NK cell (TF_NK_ = 10.78 ± 4.5%; SH_NK_ = 1.85 ± 0.4%; *p*=0.006, Figure 1(a)) and Treg (TF_Treg_ = 21.09 ± 5.12%; SH_Treg_ = 7.25 ± 0.23%; *p*=0.0011, Figure 1(b)) percentages were significantly increased in the spleens of TF tumor-bearing versus SH mice. Also, the exhaustion status of T cells was significantly elevated in the spleens of tumor-bearing compared to SH mice (TF_TCES_ = 18.23 ± 4.58; SH_TCES_ = 0.16 ± 0.054, *p* = 0.0001, Figure 1(c)). Interestingly, there was also an increase in the percentage of CTLA-4^+^ CTLs in tumor-bearing mice that was trending toward statistical significance (*p*=0.014). Importantly, the overall population percentages of CTLs and Ths were statistically equivalent (*p* > 0.0083). For Panel 4, the overall percentage of MLCs within the tumor-bearing mouse spleens was significantly elevated compared to SH (TF_MLC_ = 5.27 ± 1.11%; SH_MLC_ = 1.39 ± 0.4%, *p*=0.00031, Figure 2(a)). However, the percent populations of both monocytes/macrophages and granulocytes from tumor-bearing mouse spleens versus SH were decreased and trending toward statistical significance (*p*=0.036  and  0.014, respectively), potentially suggesting a percent increase of immature MLCs exhibiting intermediate phenotypes. Strikingly, the M1 markers Cxcl9 and Nos2 and M2 marker Tgm2 were significantly upregulated in the macrophage populations of tumor-bearing mice (*p*=0.00052 (Figure 2(b)), 4.2E-07 (Figure 2(c)), and 0.0025 (Figure 2(d)), respectively), displaying no clear transition in terms of polarization. The M2 marker Arg1 did not stain well in any of the populations examined. The statistics for each panel are summarized in Tables 5 and 6 for Panels 3 and 4, respectively.

Out of the thirteen cell populations compared between TF tumor-bearing and SH groups, seven were found to be statistically significant. On the lymphoid side, we saw significant increases in both NK cells and Tregs; this increase in splenic Tregs has been observed across multiple tumor types in preclinical models, correlates with tumor size, and is generally associated with an overall state of immune suppression [52, 53]. The splenic myeloid compartment of tumor-bearing mice was altered both in terms of MLC pool and macrophage maturation, which reflects a situation of increased inflammatory monocyte recruitment that may act as a reservoir for fast deployment to peripheral tissues during disease processes [54]. Additionally, it may also represent an accumulation of immature myeloid cells with immunosuppressive phenotypes, as suggested for other tumor models [55]. The status of macrophage activation was also significantly altered in tumor-bearing mice. Interestingly, the expression of macrophage M1 markers Nos2/Cxcl9 and M2 marker Tgm2 all increased significantly, with no clear M1/M2 bias. These data support the evolving hypothesis that macrophages with intermediate phenotypes predominate in microenvironments associated with complex tumorigenic processes and that simple M1/M2 classification may lead to gross oversimplifications that lead to incorrect conclusions. Overall, these data suggest that there is a systemic dysregulation of lymphocyte and myeloid populations created by malignancy, which can be monitored using these panels we described. The next step in this process is to more clearly define the specific functions that are associated with the observed phenotypes and what clinical outcomes they associate with, which may be used to help determine extent of disease progression and inform prognostics.

### 3.4. The Spleen Can Be Used as a Barometer of the Systemic Immune Response to Treatment with Monoclonal Antibody and Immunological Checkpoint Blocker α-PD-L1

**Figure 3:**
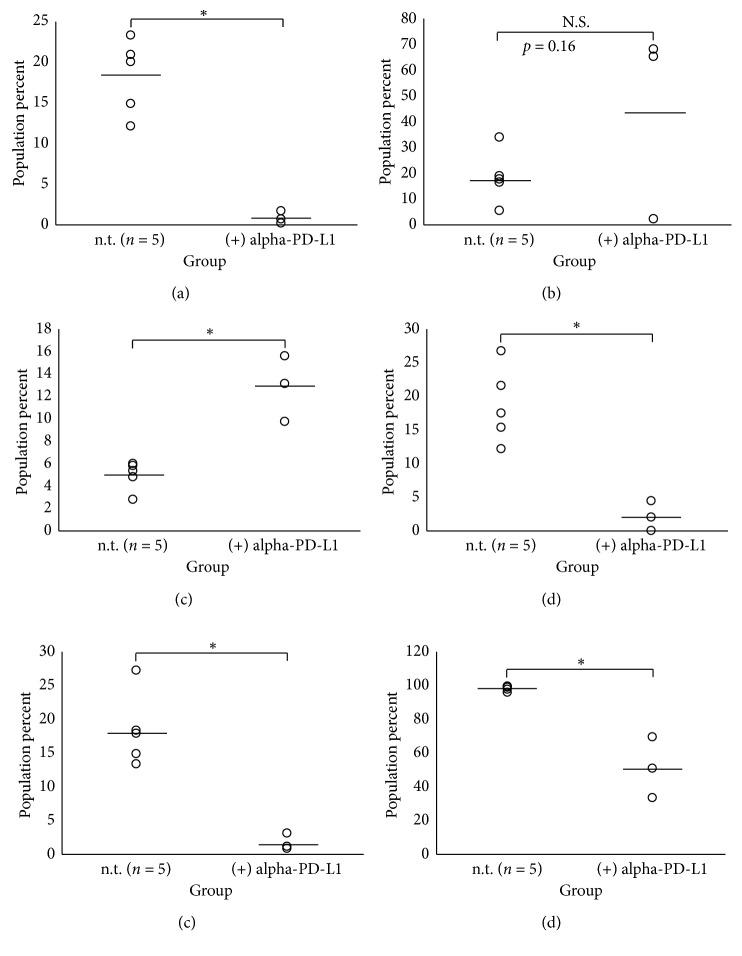
Treatment with *α*-PD-L1 significantly reverses osteosarcoma-induced immunomodulation in LLC and MLC populations in the spleens of tumor-bearing mice. Spleens were harvested at time of metastatic disease (approximately 11 weeks) after inoculation and analyzed via flow cytometry for LLC and MLC immunophenotyping from *n* = 5 (−) *α*-PD-L1 and *n* = 3 (+) *α*-PD-L1 TF tumor-bearing mice using Antibody Panels 3 (mod) and 4, respectively. For treated mice, 20 *μ*g of *α*-PD-L1 was given in phosphate buffered saline via intraperitoneal injection twice a week (Mondays and Thursdays) starting when tumors were first palpable. The data spreads of (a) TCES and (b) CTLA-4^+^ CTLs from Antibody Panel 3 (mod) are shown with significance being assigned to those populations presenting *p* value < 0.01 from a two-tailed Student's *T*-test with Bonferroni correction and 95% confidence as denoted by an asterisk. The data spreads of (c) monocytes/macrophages, (d) Tgm2^+^ macrophages, (e) Cxcl9^+^ macrophages, and (f) Nos2^+^ macrophages from Antibody Panel 4 are shown with significance being assigned to those populations presenting *p* value < 0.00714 from a two-tailed Student's *T*-test with Bonferroni correction and 95% confidence as denoted by an asterisk. PD-L1: programmed death-ligand; LLC: lymphoid lineage cell; MLC: myeloid lineage cell; TF: transfected; TCES: T-cell exhaustion status; CTLA-4: cytotoxic T-lymphocyte-associated protein 4; CTL: cytotoxic T lymphocyte; Tgm2: transglutaminase 2; Cxcl9: chemokine (C-X-C motif) ligand 9; Nos2: nitric oxide synthase 2.

In Section 3, we determined that our antibody panels could accurately characterize malignancy-induced immunomodulations occurring alongside disease progression. As such, if we are able to assess how the immune system is changing in response to immunotherapeutics in real-time, we may be able to adapt treatment regimens so that the appropriate immune responses are elicited in the right way at the right time on a patient-by-patient basis. Therefore, if patients' systemic immune systems are sampled regularly, important tumor immune escape mechanisms such as these may be exposed and properly accounted for therapeutically. Therefore, we sought to determine whether the spleen could be used as a systemic immunologic barometer to assess immunotherapy-induced microenvironment changes. The immunotherapy chosen for this portion of the study was *α*-PD-L1, which is used in different cancers with varying success rates [6]. As *α*-PD-L1 blockade activity is already well-established in the literature [56, 57] and overall treatment efficacy was not the desired outcome of the study, no isotype controls were deemed necessary. For these studies, TF tumor-bearing mice (*n* = 3) were treated intraperitoneally with 20 *μ*g of *α*-PD-L1 antibody in PBS twice a week starting at the first palpable sign of primary lesion. Primary tumors were removed at approximately 1.7 cm^3^, and mice were euthanized at approximately 11 weeks after inoculation. Harvested spleens were subjected to a modified (mod) Panel 3 (lacking the NKp46 NK cell identifier) and Panel 4. Using the Bonferroni correction, individual comparisons to assess significance between *α*-PD-L1 treated (*n* = 3) and untreated (*n* = 5) TF tumor-bearing mice were made via Student's *T*-test using *α* = 0.01 and *α* = 0.00714 for Antibody Panels 3 (mod) and 4 at the 95% confidence interval, respectively. Of those populations investigated in Panel 3 (mod), *α*-PD-L1 treatment was shown to significantly reduce splenic TCES (TF_(−*α*PD-L1)_ = 18.23 ± 4.58; TF_(+*α*PD-L1)_ = 0.79 ± 0.71, *p*=0.00072) as shown in Figure 3(a). Interestingly, however, two out of the three mice that showed decreased TCES in response to *α*-PD-L1 therapy also showed a large increase in the percentage of CTLA-4^+^ expressed on CTLs (Figure 3(b)), perhaps suggestive of a potential escape mechanism by which tumors can depress T-cell activity. *α*-PD-L1 treatment also greatly increased the percent population of monocytes/macrophages (Figure 3(c): TF_(−*α*PD-L1)_ = 4.92 ± 1.29%; TF_(+*α*PD-L1)_ = 12.8 ± 2.92%, *p*=0.0016) and decreased the percentage of macrophages expressing Tgm2 (Figure 3(d): TF_(−*α*PD-L1)_ = 18.58 ± 5.69%; TF_(+*α*PD-L1)_ = 2.1 ± 2.17%, *p*=0.0034), Cxcl9 (Figure 3(e): TF_(−*α*PD-L1)_ =18.28 ± 5.38%; TF_(+*α*PD-L1)_ = 1.52 ± 1.24%, *p*=0.002), and Nos2 (Figure 3(f): TF_(−*α*PD-L1)_ = 97.99 ± 1.62%; TF_(+*α*PD-L1)_ = 50.89 ± 18.07%, *p*=0.00086), essentially normalizing the M1/M2 macrophage polarization distribution back to SH status. Complete data summaries can be found in Tables 7 and 8 for Panels 3 (mod) and 4, respectively.

Immunotherapies that include PD-1/PD-L1 blockade have shown prolonged clinical activity against various human malignancies [58–60] excluding osteosarcoma [10, 61], despite the fact that there is evidence that PD-L1 contributes to osteosarcoma disease progression [33, 34]. Therefore, *α*-PD-L1 was chosen as our quintessential immunotherapeutic for the dual purposes of testing the ability of our antibody panels to detect therapy-induced changes in splenic microenvironment and to potentially shed some light on the immunologic mechanisms behind its therapeutic shortcomings. Interestingly, we found that while both *α*-PD-L1 treated and nontreated mice eventually succumbed to disease, *α*-PD-L1 monotherapy alone was sufficient to substantially reverse many immunological phenomena observed in nontreated tumor-bearing mice. However, while individual M1 and M2 markers between treated and untreated groups did show significant changes, the M1 : M2 marker ratios were statistically equivalent (*p*=0.21). The data suggest that *α*-PD-L1 monotherapy alone can stabilize but not induce generalized M1-biased macrophage polarization that has been associated with decreased metastasis [62]. Still, these data offer a possible explanation for why combination therapies including *α*-PD-L1 therapy are more effective [61], as it appears to neutralize many of the immunological effects induced by malignancy. Interestingly, two out of three mice treated with *α*-PD-L1 therapy showed drastically increased expression of CTLA-4 on CTLs, revealing perhaps a compensatory mechanism by which tumors can circumnavigate *α*-PD-L1 monotherapeutic intervention which supports previous findings [61]. Taken together, these data argue for the use of immunotherapeutic “cocktails” that target multiple pathways similar to the approach taken for human immunodeficiency virus/acquired immune deficiency syndrome (HIV/AIDS) therapy. Furthermore, we have provided a reasonable explanation for why *α*-PD-L1 monotherapy for osteosarcoma has been less successful in the past. As such, we propose that the spleen may serve as a barometer for real-time monitoring of disease progression and patient response to immunotherapy and deserves further investigation.

## 4. Conclusions

Introduction of a nonviral luciferase reporter vector into K7M2 osteosarcoma tumor cells had no significant effect on the local or systemic immune responses to disease progression and metastasis in an orthotopic murine model of osteosarcoma, confirming its utility for accurately modeling complex immune microenvironment dynamics despite the luciferase reporter transfection. Our model can be used to accurately assess both disease burden (via IVIS) and immune dynamics with confidence that the reporter protein will not cause significant immunological disturbances. Additionally, it was shown that osteosarcoma disease progression and metastasis induce systemic dysregulation of immune cell populations including macrophage maturation and M1/M2 marker expression, TCES, and NK cell percent populations when compared to disease-free SH controls. These data suggest that osteosarcoma-induced immunomodulation is observable in the spleen with the antibody panels described and offer new insight into the complex roles that macrophages play in osteosarcoma disease progression. Indeed, both the prevalence of tumor-associated macrophages and expression of single macrophage M1/M2 polarization markers have been previously associated with disease events that when taken together seem contradictory [13, 26, 62, 63]. In this study, we showed that osteosarcoma disease progression induces clear dysregulation of macrophage activation with increased expression of both M1 and M2 markers, offering further proof as to why single marker M1/M2 macrophage identifiers do not accurately assess polarization status in complex malignant settings. Our studies also showed that low-dose *α*-PD-L1 therapy alone normalized osteosarcoma-induced systemic immunodysregulation back to that of SH status without changing clinical outcome; however, a compensatory increase in the immunosuppressive marker CTLA-4 on CTLs was observed following PD-L1 blockade indicating a potential tumor escape mechanism. These data suggest that immune escape mechanisms like PD-L1/CTLA-4 counter-regulation may be uncovered by immunophenotyping systemic immune organs like the spleen, which could be extremely useful clinically by adapting therapies to the immunophenotype that is occurring at a specific time point.

## Figures and Tables

**Table 1:** Specific cell populations discussed and their corresponding defining markers.

Cell population	Defining markers
Tumor cells and stroma	CD45^−^PD-L1^±^
Cytotoxic T lymphocyte	CD45^+^CD8^+^
T helper lymphocyte	CD45^+^CD4^+^
Myeloid lineage cell	CD45^+^CD11 b^+^
Granulocyte	CD45^+^CD11 b^+^Ly6G^+^Ly6C^−^
Monocyte/macrophage	CD45^+^CD11 b^+^Ly6C^+^Ly6G^−^
Exhausted cytotoxic T lymphocyte	CD45^+^CD8^+^ PD-1^±^Tim-3^±^
Exhausted T helper lymphocyte	CD45^+^CD4^+^PD-1^±^Tim-3^±^
CTLA-4-expressing cytotoxic T lymphocyte	CD45^+^CD8^+^CTLA-4^+^
M1-like macrophage	Cxcl9^±^Nos2^±^
M2-like macrophage	Arg1^±^Tgm2^±^

PD-L1 = programmed death-ligand 1; PD-1 = programmed cell death protein 1; Tim-3 = T-cell immunoglobulin and mucin-domain containing-3; CTLA-4^+^ = cytotoxic T-lymphocyte-associated protein 4; Cxcl9 = chemokine (C-X-C) motif ligand 9; Nos2 = nitric oxide synthase 2; Arg1 = arginase 1; Tgm2 = transglutaminase 2.

**Table 2:** Population distribution comparisons of WBCs isolated from primary tumors of BALB/c mice inoculated with TF (*n* = 15) or WT (*n* = 11) K7M2 cells.

Population	WT % expression	TF % expression	*p*
NK cells	19.76 ± 4.76%	20.94 ± 12.67%	0.77
CTLs	5.45 ± 3.09%	6.07 ± 7.55%	0.8
Ths	6.96 ± 1.69%	5.42 ± 3.39%	0.18
Tregs	16.95 ± 10.44%	24.02 ± 15.37%	0.2
MLCs	36.5 ± 23.12%	34.74 ± 17.53%	0.95
Granulocytes	35.8 ± 18.32%	36.59 ± 16.38%	0.91

The statistics for each group are summarized accordingly with significance being assigned to those populations presenting *p* value < 0.0083 from a two-tailed Student's *T*-test with Bonferroni correction and 95% confidence as denoted by an asterisk. WBC = white blood cell; TF = transfected; WT = wild-type; NK = natural killer; CTL = cytotoxic T lymphocyte; Th = T helper cell; Treg = T regulatory cell; MLC = myeloid lineage cell.

**Table 3:** Non-WBC cell PD-L1 expression of primary tumors isolated from BALB/c mice inoculated with TF (*n* = 15) or WT (*n* = 11) K7M2 cells.

Marker	WT % expression	TF % expression	*p*
PD-L1	1.96 ± 0.77%	1.91 ± 1.28%	0.9

Statistical significance was assessed using a two–tailed Student's *T*-test and 95% confidence. WBC = white blood cell; PD-L1 = programmed death-ligand 1; TF = transfected; WT = wild-type.

**Table 4:** Population distribution comparison of WBCs isolated from the spleens of BALB/c mice inoculated with TF (*n* = 9) or WT (*n* = 10) K7M2 cells.

Population	WT % expression	TF % expression	*p*
NK cells	5.35 ± 4.58%	7.55 ± 5%	0.33
CTLs	7.23 ± 2.41%	5.03 ± 2.64%	0.08
Ths	18 ± 13.61%	11.71 ± 12.95%	0.32
Tregs	12.68 ± 6.01%	16.19 ± 8.43%	0.31
MLCs	5.2 ± 11.41%	9.76 ± 10.76%	0.38
Granulocytes	15.27 ± 21.82%	52.33 ± 19.02%	0.0011^*∗*^

The statistics for each group are summarized accordingly with significance being assigned to those populations presenting *p* values < 0.0083 from a two-tailed Student's *T*-test with Bonferroni correction and 95% confidence as denoted by an asterisk. WBC = white blood cell; TF = transfected; WT = wild-type; NK = natural killer; CTL = cytotoxic T lymphocyte; Th = T helper cell; Treg = T regulatory cell; MLC = myeloid lineage cell.

**Table 5:** Population distribution comparison of LLCs isolated from the spleens of mice from TF tumor-bearing (*n* = 5) and SH (*n* = 4) groups.

Population	TF % expression/TCES	SH % expression/TCES	*p*
NK cells	10.78 ± 4.5%	1.85 ± 0.4%	0.006^*∗*^
CTLs	2.73 ± 1.22%	3.72 ± 0.86%	0.21
Ths	6.45 ± 3.08%	7.72 ± 1.1%	0.46
Tregs	21.09 ± 5.12%	7.25 ± 0.23%	0.0011^*∗*^
CTLA-4^+^ CTLs	18.35 ± 10.12%	1.72 ± 0.42%	0.014
TCES	18.23 ± 4.58	0.16 ± 0.054	0.0001^*∗*^

The statistics for each group are summarized accordingly with significance being assigned to those populations presenting *p* values < 0.0083 from a two-tailed Student's *T*-test with Bonferroni correction and 95% confidence as denoted by an asterisk. LLC = lymphoid lineage cell; TF = transfected; NK = natural killer; CTL = cytotoxic T lymphocyte; Th = T helper cell; Treg = T regulatory cell; CTLA-4^+^ CTL = cytotoxic T-lymphocyte-associated protein 4 positive cytotoxic T lymphocyte; TCES = T-cell exhaustion status.

**Table 6:** Population distribution comparison of MLCs isolated from the spleens of mice from TF tumor-bearing (*n* = 5) and SH (*n* = 4) groups.

Population	TF % expression	SH % expression	*p*
MLCs	5.27 ± 1.11%	1.39 ± 0.4%	0.00031^*∗*^
Monocytes/macrophages	4.92 ± 1.29%	7.49 ± 1.7%	0.036
Granulocytes	57.49 ± 9.62%	74.69 ± 4.55	0.014
Tgm2^+^ macrophages	18.58 ± 5.69%	4.22 ± 2.66%	0.0025^*∗*^
Arg1^+^ macrophages	0.3 ± 0.56%	0 ± 0%	0.32
Cxcl9^+^ macrophages	18.28 ± 5.38%	1.44 ± 1.23%	0.00052^*∗*^
Nos2^+^ macrophages	97.99 ± 1.62%	16.56 ± 10.19%	4.2E-07^*∗*^

The statistics for each group are summarized accordingly with significance being assigned to those populations presenting *p* values < 0.00714 from a two-tailed Student's *T*-test with Bonferroni correction and 95% confidence as denoted by an asterisk. MLC = myeloid lineage cell; TF = transfected; Tgm2 = transglutaminase 2; Arg1 = arginase 1; Cxcl9 = chemokine (C-X-C) motif ligand 9; Nos2 = nitric oxide synthase 2.

**Table 7:** Treatment with *α*-PD-L1 significantly reduces TCES but does not affect parent populations of LLCs in the spleens of TF tumor-bearing mice.

Population	TF (−) *α*-PD-L1 % expression/TCES	TF (+) *α*-PD-L1 % expression/TCES	*p*
CTLs	2.73 ± 1.22%	2.85 ± 1.13%	0.9
Ths	6.45 ± 3.08%	5.14 ± 3.74%	0.61
Tregs	21.09 ± 5.12%	26.63 ± 7.02%	0.24
CTLA-4^+^ CTLs	18.35 ± 10.12%	44.95 ± 37.19%	0.16
TCES	18.23 ± 4.58	0.79 ± 0.71	0.00072^*∗*^

Spleens were harvested at approximately 11 weeks post-inoculation and the tissue was subjected to Panel 3 (mod) for immune cell population analysis from *n* = 5 (−) *α*-PD-L1 and *n* = 3 (+) *α*-PD-L1 TF tumor-bearing mice. For treated mice, 20 *μ*g of *α*-PD-L1 was given in PBS via intraperitoneal injection twice a week starting when tumors were first palpable. The statistics for each group are summarized accordingly with significance being assigned to those populations presenting *p* values < 0.01 from a two-tailed Student's *T*-test with Bonferroni correction and 95% confidence as denoted by an asterisk. PD-L1 = programmed death-ligand 1; TF = transfected; LLC: lymphoid lineage cell; CTL = cytotoxic T lymphocyte; Th = T helper cell; Treg = T regulatory cell; CTLA-4^+^ CTL = cytotoxic T-lymphocyte-associated protein 4 positive cytotoxic T lymphocyte; TCES = T-cell exhaustion status.

**Table 8:** Treatment with *α*-PD-L1 dramatically affects macrophage maturation and M1/M2 polarization in the spleens of TF tumor-bearing mice.

Population	TF (−) *α*-PD-L1 % expression	TF (+) *α*-PD-L1 % expression	*p*
MLCs	5.27 ± 1.11%	5.73 ± 1.68%	0.65
Monocytes/macrophages	4.92 ± 1.29%	12.8 ± 2.92%	0.0016^*∗*^
Granulocytes	57.49 ± 9.62%	51.37 ± 9.11%	0.41
Tgm2^+^ macrophages	18.58 ± 5.69%	2.1 ± 2.17%	0.0034^*∗*^
Arg1^+^ macrophages	0.3 ± 0.56%	0.23 ± 0.17%	0.84
Cxcl9^+^ macrophages	18.28 ± 5.38%	1.52 ± 1.24%	0.002^*∗*^
Nos2^+^ macrophages	97.99 ± 1.62%	50.89 ± 18.07%	0.00086^*∗*^

Spleens were harvested at approximately 11 weeks post-inoculation and the tissue was subjected to Panel 4 for immune cell population analysis from *n* = 5 (−) *α*-PD-L1 and *n* = 3 (+) *α*-PD-L1 TF tumor-bearing mice. For treated mice, 20 *u*g of *α*-PD-L1 was given in PBS via intraperitoneal injection twice a week starting when tumors were first palpable. The statistics for each group are summarized accordingly with significance being assigned to those populations presenting *p* values < 0.00714 from a two-tailed Student's *T*-test with Bonferroni correction and 95% confidence as denoted by an asterisk. PD-L1 = programmed death-ligand 1; TF = transfected; MLC = myeloid lineage cell; Tgm2 = transglutaminase 2; Arg1 = arginase 1; Cxcl9 = chemokine (C-X-C) motif ligand 9; Nos2 = nitric oxide synthase 2.

## Data Availability

The primary flow cytometry data for this manuscript can be accessed with no restrictions by contacting the primary author.

## Supplementary Materials

Supplementary Materials Supplementary Figure 1: example gating schema for myeloid lineage cells, monocytes/macrophages, and granulocytes from Antibody Panel 1. Cells were gated into CD45^+^ and CD45^−^ groups; from there, CD4^−^CD8^−^CD11 b^+^ cells myeloid lineage cells were gated into Ly6C^+^Ly6G^−^ macrophages/monocytes and Ly6G^+^Ly6C^−^ granulocytes. Supplementary Figure 2: example gating schema for T helper cells, cytotoxic T cells, and T regulatory cells from Antibody Panel 1. Cells were gated into CD45^+^ and CD45^−^ groups; from there, cells were gated into CD4^+^ T helper cells and CD8^+^ cytotoxic T cells. CD4^+^ cells were further gated using Foxp3^+^ as a defining marker of T regulatory cells. Supplementary Figure 3: example gating schema for natural killer cells from Antibody Panel 1. Cells were gated into CD45^+^ and CD45^−^ groups; from there, CD45^+^ cells were gated into NKp46^+^ natural killer cells. Supplementary Figure 4: example gating schema for PD-L1 positive non-white blood cell tumor cells from Antibody Panel 2. Tumor cells were gated into CD45^+^ and CD45^−^ groups; from there, CD45^−^ non-white blood cell tumor cells were assessed for programmed death-ligand 1 (PD-L1) positivity. Note that, although the figure displays staining for CD95 positivity, this marker was not included in the analysis for PD-L1 expression in the manuscript. Supplementary Figure 5: example gating schema for cytotoxic T cells, T helper cells, natural killer cells, and T regulatory cells from Antibody Panel 3. Cells were gated into CD45^+^ and CD45^−^ groups; from there, CD45^+^ cells were gated into NKp46^+^ natural killer cells, CD45^+^CD8^+^ cytotoxic T cells, and CD45^+^CD4^+^ T helper cells. CD45^+^CD4^+^ T cells were further gated for Foxp3 positivity to define CD45^+^CD4^+^Foxp3^+^ T regulatory cells. Supplementary Figure 6: example gating schema for T-cell exhaustion and functional status from Antibody Panel 3. CD4^+^ T helper cells and CD8+ cytotoxic T cells were assessed for expression of the T-cell exhaustion markers programmed cell death protein 1 (PD-1) and T-cell immunoglobulin and mucin-domain containing-3 (Tim-3). CD8^+^ T cells were also separately assessed for expression of inhibitory molecule cytotoxic T lymphocyte-associated protein 4 (CTLA-4). Supplementary Figure 7: example gating schema for myeloid lineage cells, monocytes/macrophages, and granulocytes from Antibody Panel 4. Cells were gated into CD45^+^CD11 b^+^ myeloid lineage cells; from the CD45^+^CD11 b^+^ quadrant, cells were further gated into Ly6C^+^Ly6G^−^ macrophages/monocytes and Ly6G^+^Ly6C^−^ granulocytes. Supplementary Figure 8: example gating scheme for markers of macrophage M1/M2 polarization from Antibody Panel 4. CD11 b^+^Ly6C^+^ monocytes/macrophages were assessed for expression of M1 activation markers chemokine (C-X-C motif) ligand 9 (Cxcl9) and nitric oxide synthase 2 (Nos2) and M2 activation markers transglutaminase 2 (Tgm2) and arginase 1 (Arg1).
