# Supplementary figures and images for: Using the Spleen as an In Vivo Systemic Immune Barometer Alongside Osteosarcoma Disease Progression and Immunotherapy with α-PD-L1
Source: Sarcoma. 2018 Dec 12;2018:8694397. doi: 10.1155/2018/8694397 (PMC6311869; doi:10.1155/2018/8694397)

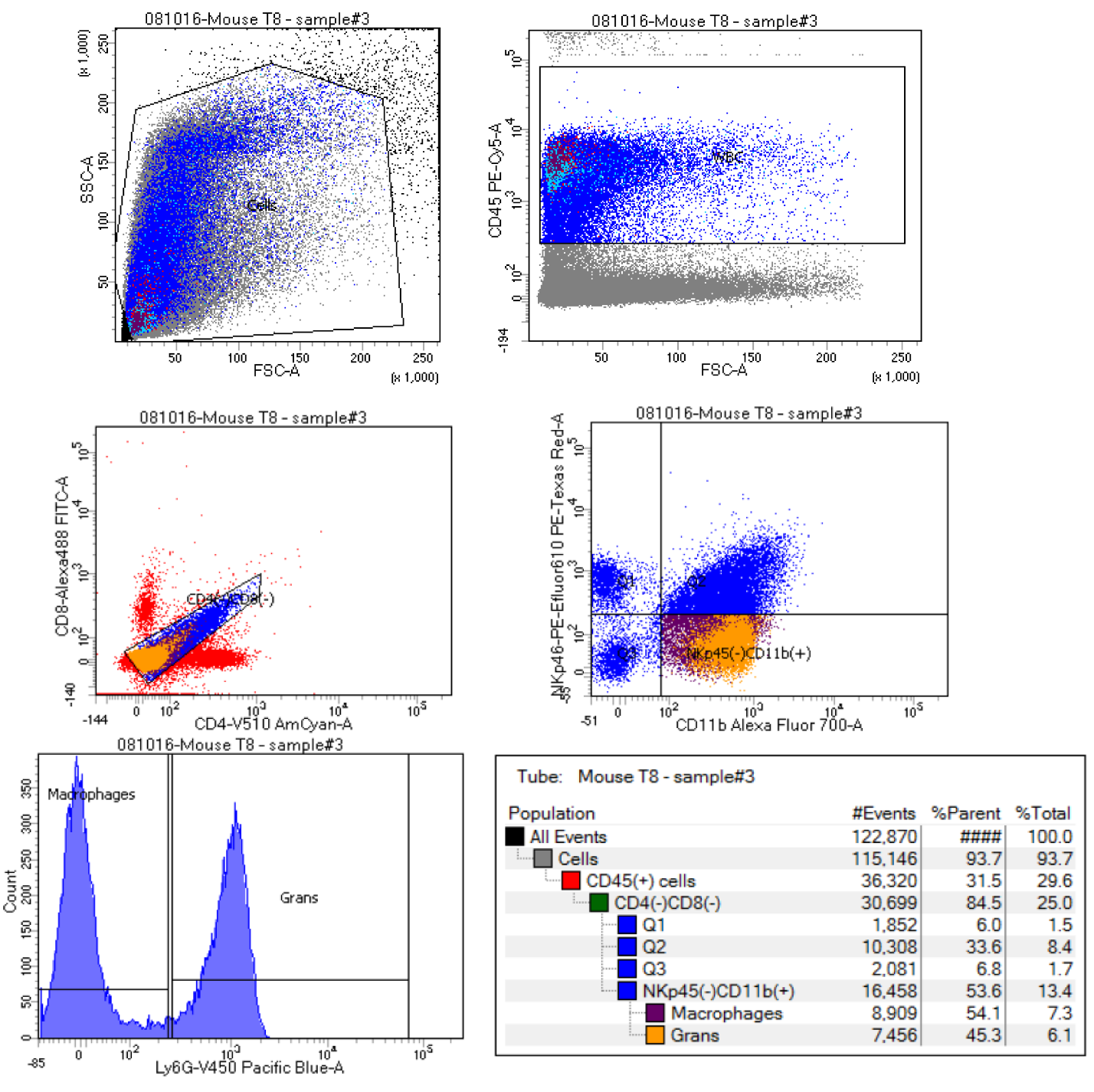


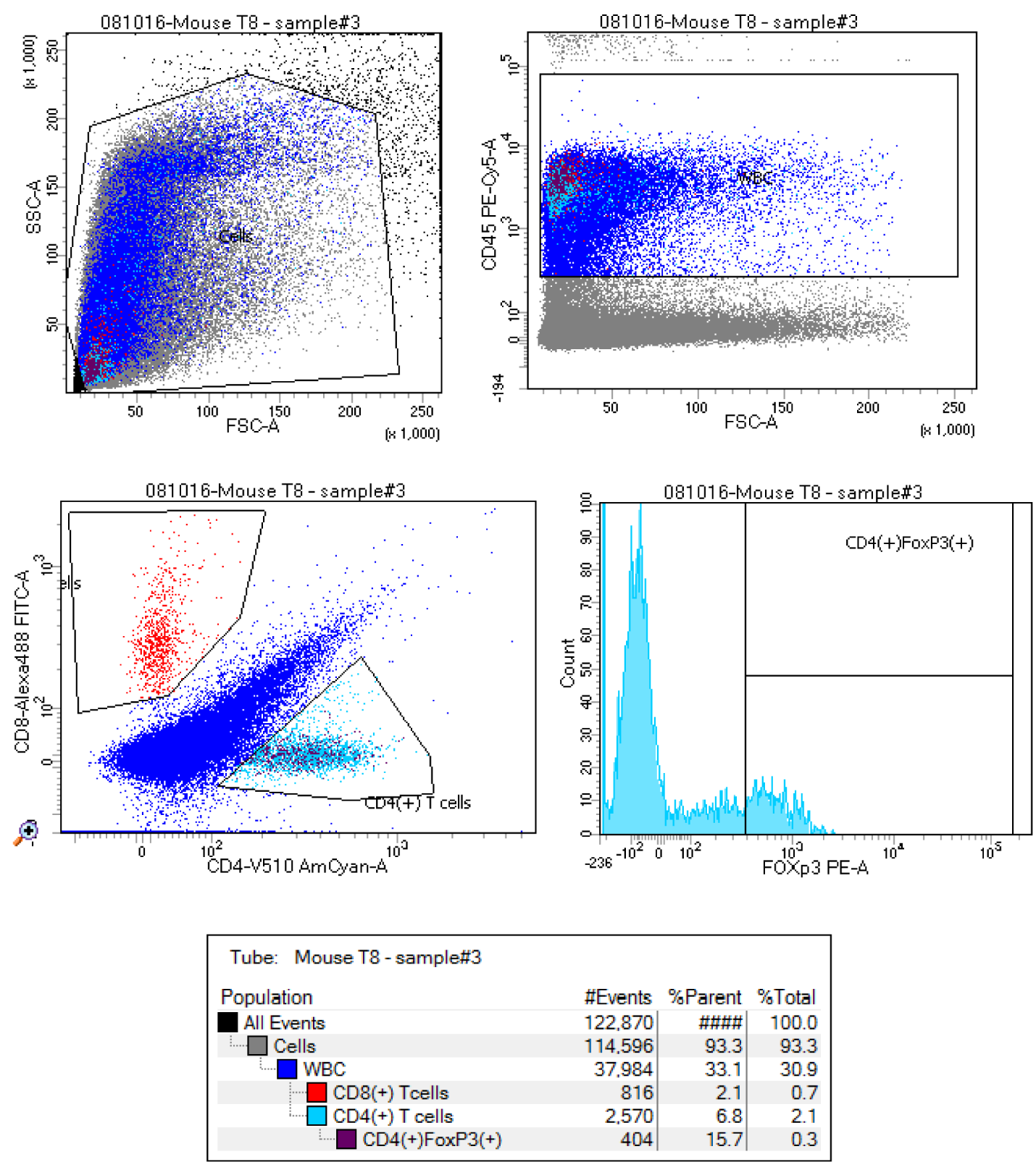


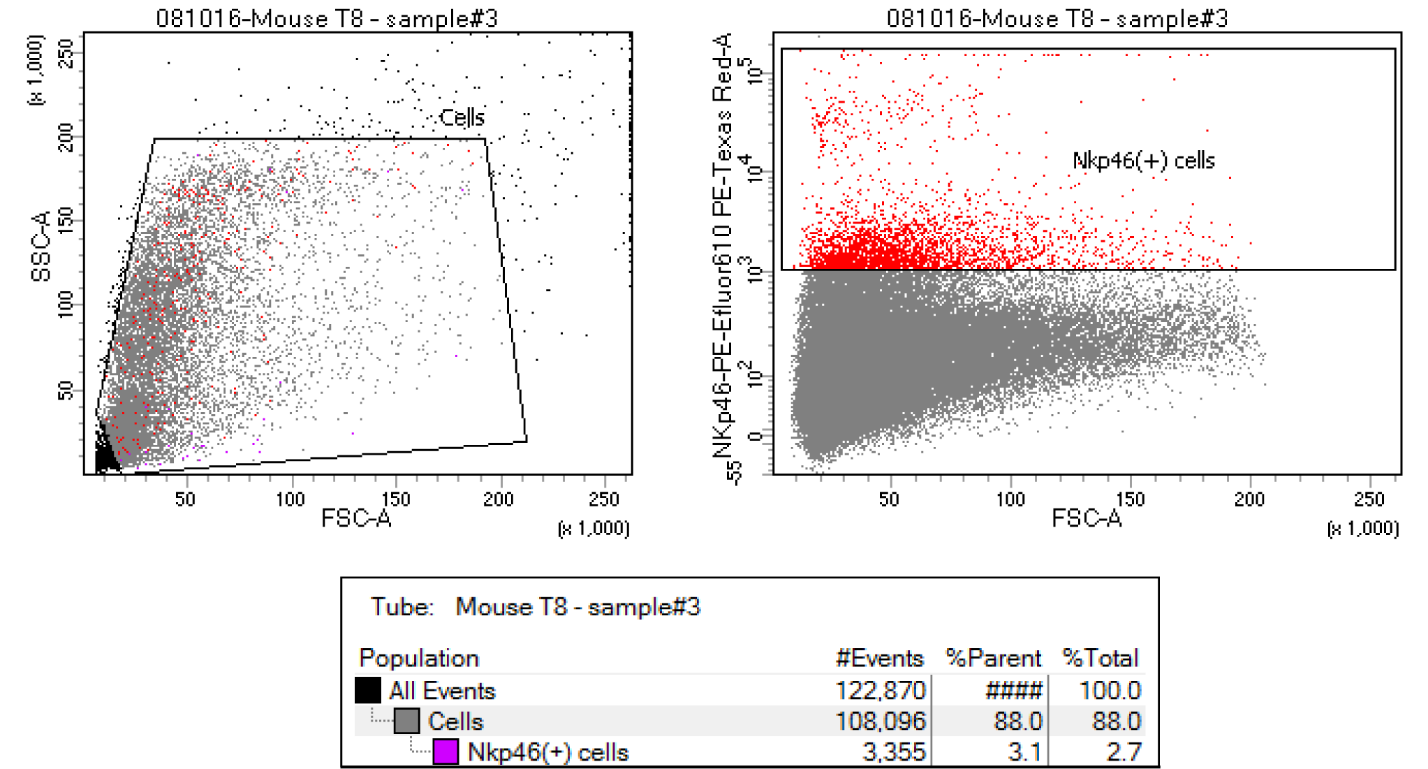


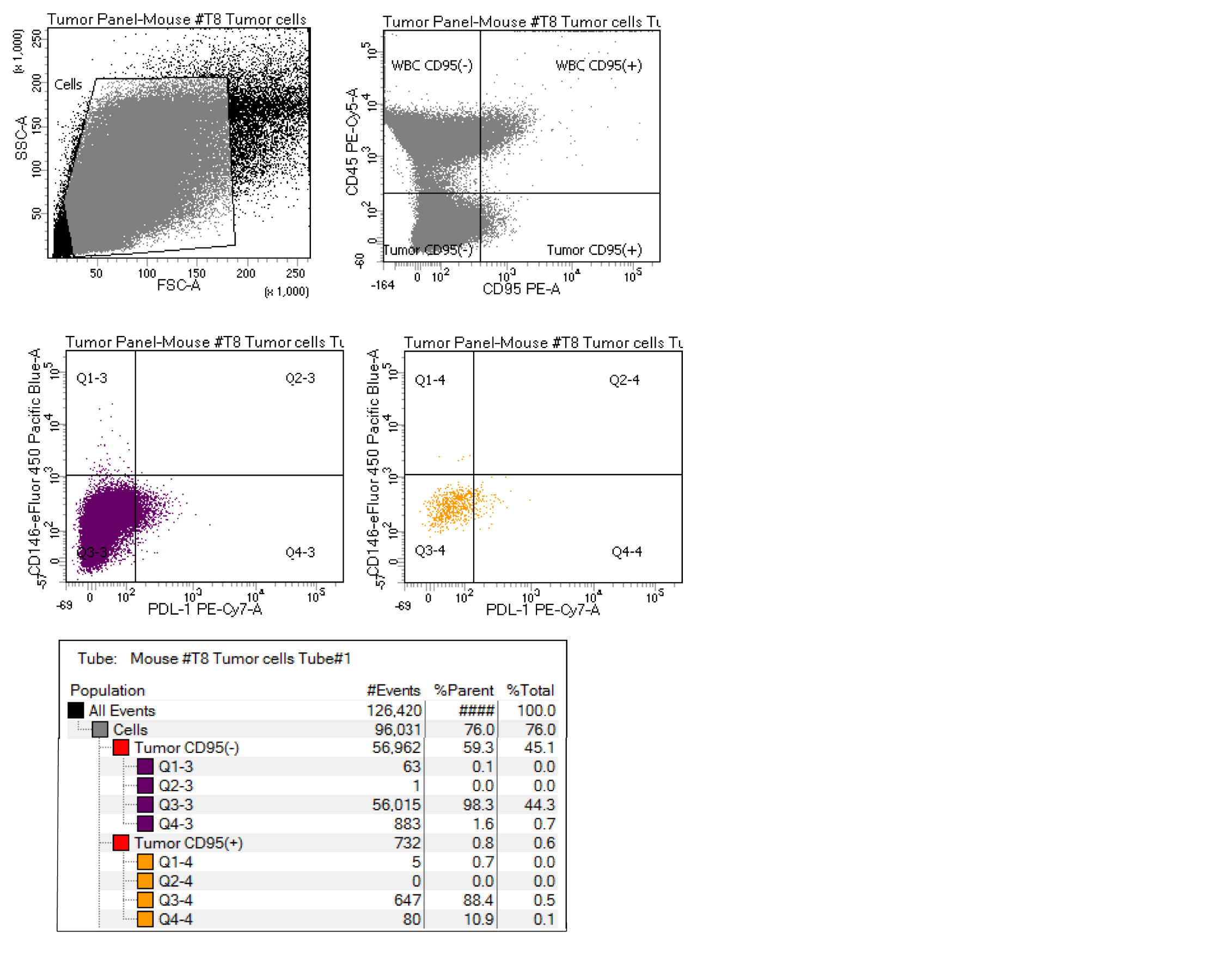


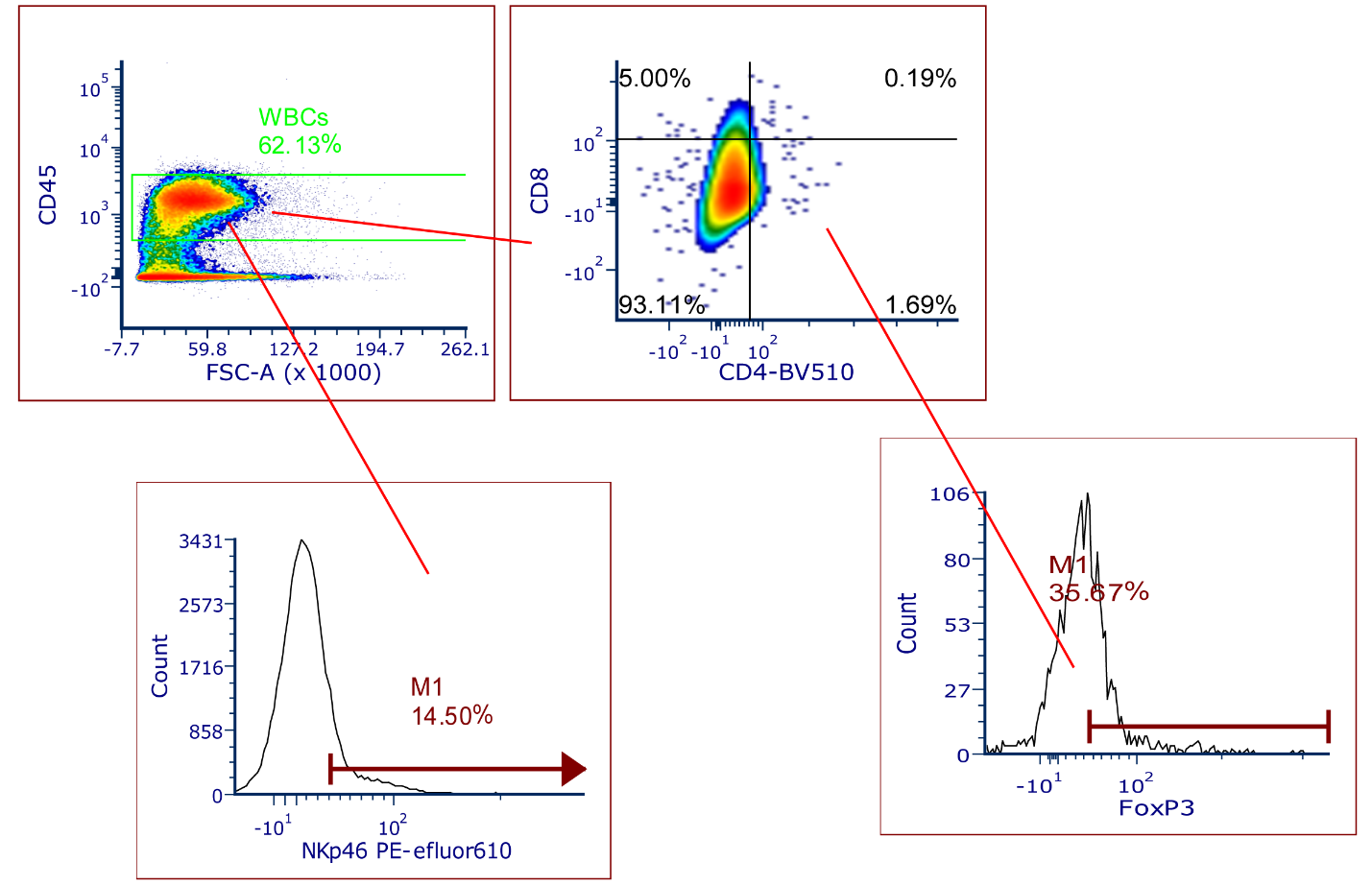


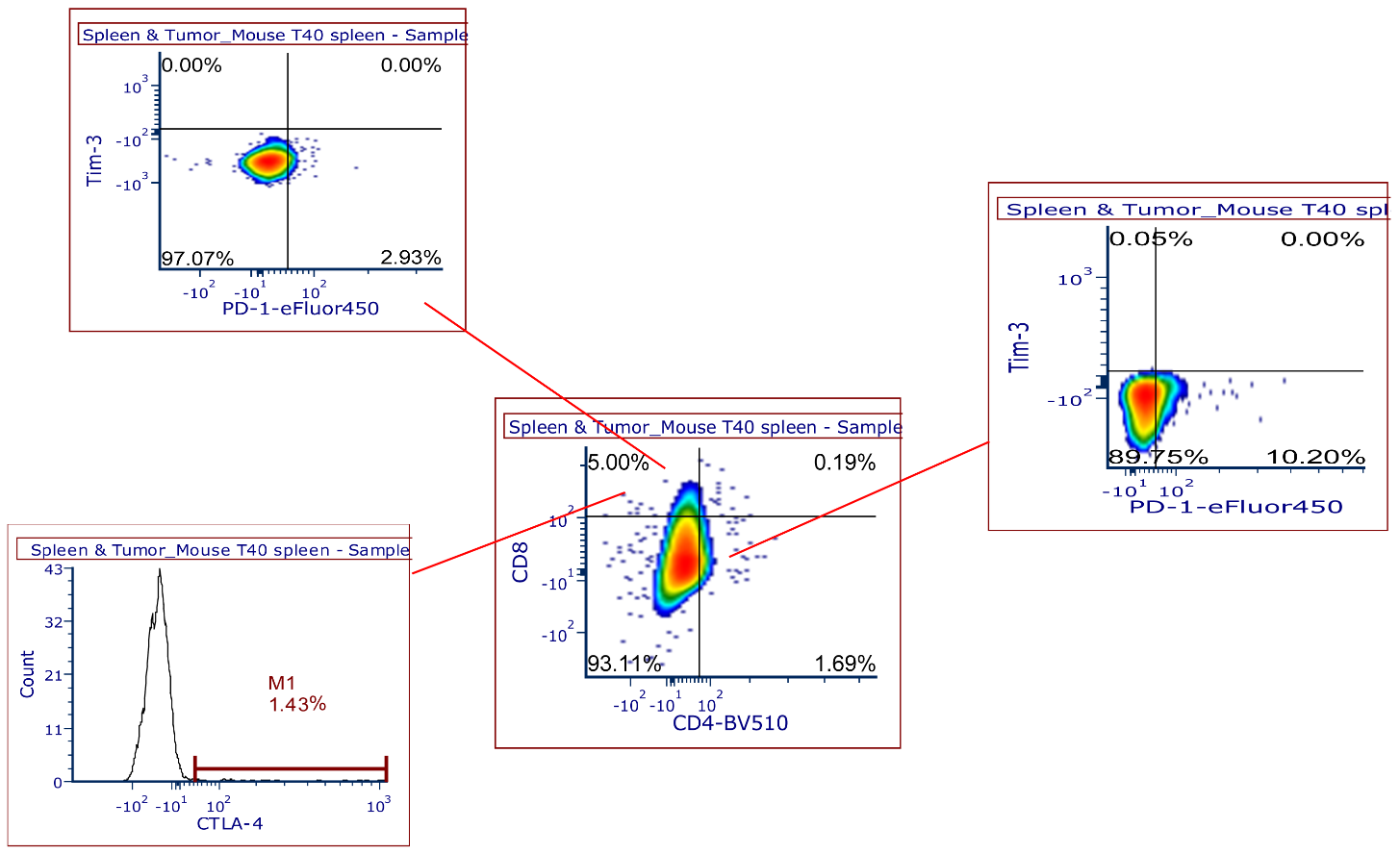


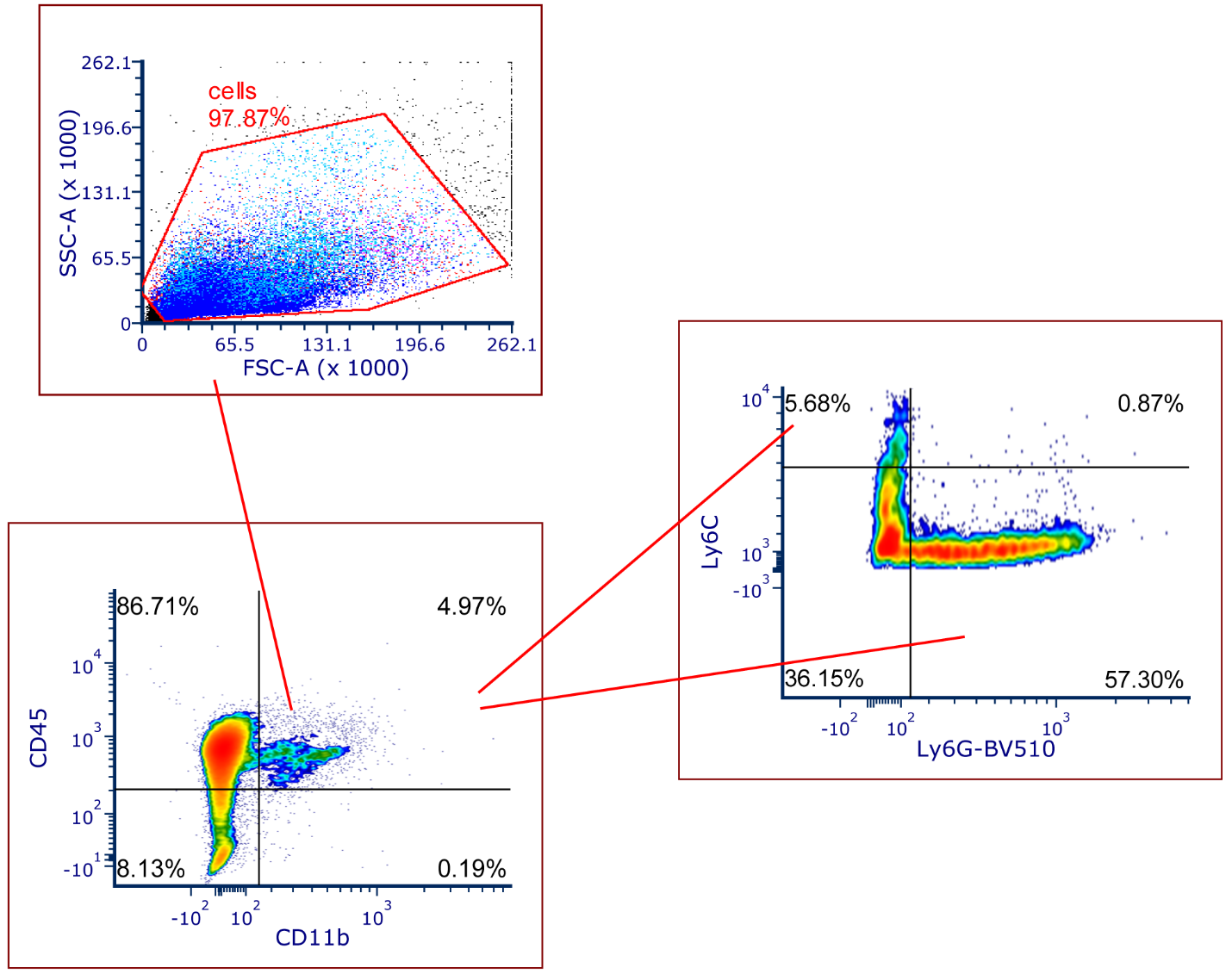


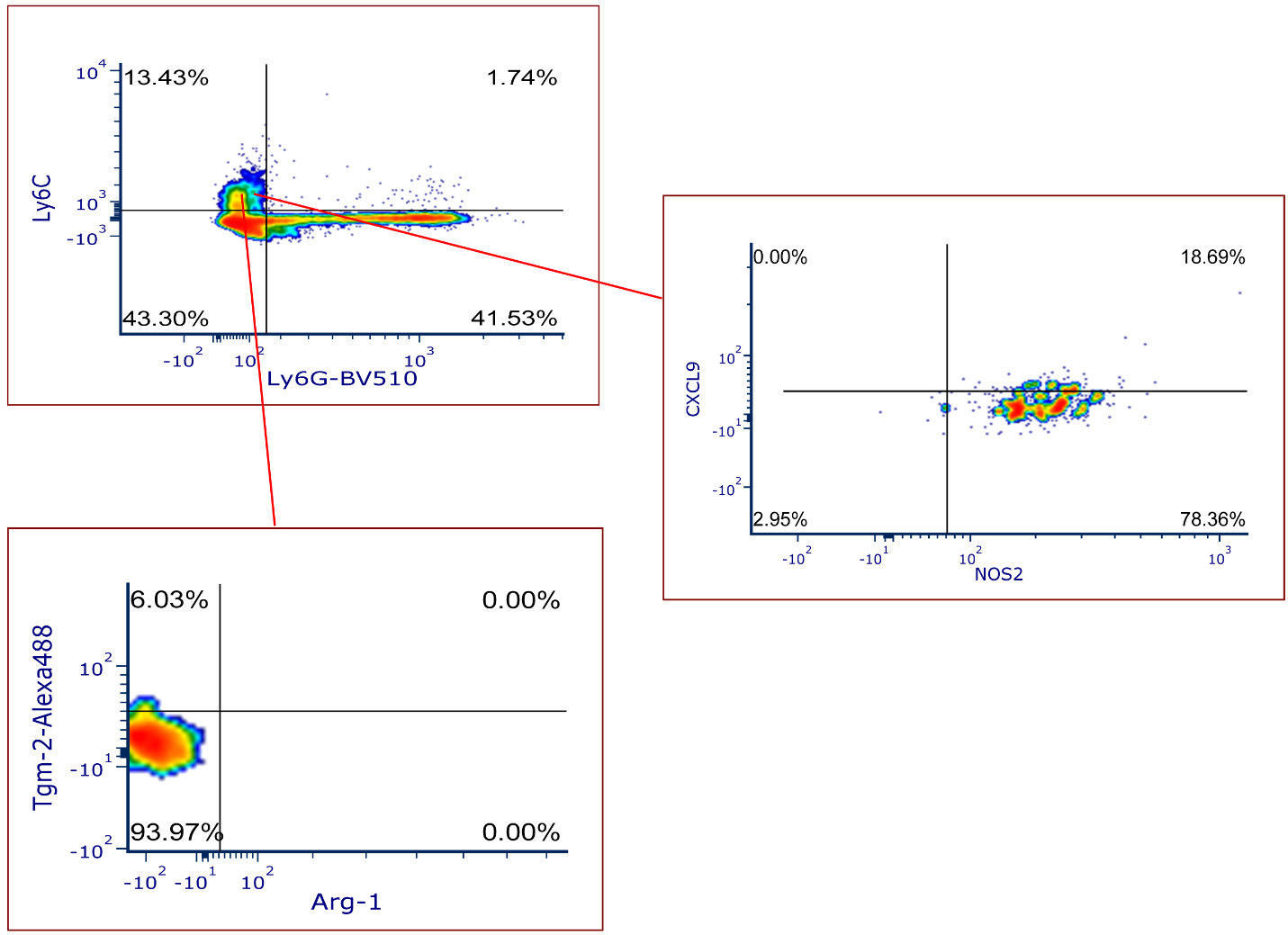

Supplement: Supplementary Materials — Supplementary Figure 1: example gating schema for myeloid lineage cells, monocytes/macrophages, and granulocytes from Antibody Panel 1. Cells were gated into CD45+ and CD45− groups; from there, CD4−CD8−CD11 b+ cells myeloid lineage cells were gated into Ly6C+Ly6G− macrophages/monocytes and Ly6G+Ly6C− granulocytes. Supplementary Figure 2: example gating schema for T helper cells, cytotoxic T cells, and T regulatory cells from Antibody Panel 1. Cells were gated into CD45+ and CD45− groups; from there, cells were gated into CD4+ T helper cells and CD8+ cytotoxic T cells. CD4+ cells were further gated using Foxp3+ as a defining marker of T regulatory cells. Supplementary Figure 3: example gating schema for natural killer cells from Antibody Panel 1. Cells were gated into CD45+ and CD45− groups; from there, CD45+ cells were gated into NKp46+ natural killer cells. Supplementary Figure 4: example gating schema for PD-L1 positive non-white blood cell tumor cells from Antibody Panel 2. Tumor cells were gated into CD45+ and CD45− groups; from there, CD45− non-white blood cell tumor cells were assessed for programmed death-ligand 1 (PD-L1) positivity. Note that, although the figure displays staining for CD95 positivity, this marker was not included in the analysis for PD-L1 expression in the manuscript. Supplementary Figure 5: example gating schema for cytotoxic T cells, T helper cells, natural killer cells, and T regulatory cells from Antibody Panel 3. Cells were gated into CD45+ and CD45− groups; from there, CD45+ cells were gated into NKp46+ natural killer cells, CD45+CD8+ cytotoxic T cells, and CD45+CD4+ T helper cells. CD45+CD4+ T cells were further gated for Foxp3 positivity to define CD45+CD4+Foxp3+ T regulatory cells. Supplementary Figure 6: example gating schema for T-cell exhaustion and functional status from Antibody Panel 3. CD4+ T helper cells and CD8+ cytotoxic T cells were assessed for expression of the T-cell exhaustion markers programmed cell death protein 1 ( [file 8694397.f1.docx]
